# The relationship between cancer and biomechanics

**DOI:** 10.3389/fonc.2023.1273154

**Published:** 2023-10-12

**Authors:** Liqi Bao, Hongru Kong, Yang Ja, Chengchao Wang, Lei Qin, Hongwei Sun, Shengjie Dai

**Affiliations:** ^1^ Department of Hepatobiliary and Pancreatic Surgery, The First Affiliated Hospital of Wenzhou Medical University, Wenzhou, Zhejiang, China; ^2^ Renji College, Wenzhou Medical University, Wenzhou, Zhejiang, China; ^3^ The First Clinical Medical College, Wenzhou Medical University, Wenzhou, Zhejiang, China; ^4^ Department of General Surgery, The First Affiliated Hospital of Soochow University, Suzhou, Jiangsu, China

**Keywords:** cancer, biomechanics, tumor microenvironment, metastasis, invasion

## Abstract

The onset, development, diagnosis, and treatment of cancer involve intricate interactions among various factors, spanning the realms of mechanics, physics, chemistry, and biology. Within our bodies, cells are subject to a variety of forces such as gravity, magnetism, tension, compression, shear stress, and biological static force/hydrostatic pressure. These forces are perceived by mechanoreceptors as mechanical signals, which are then transmitted to cells through a process known as mechanical transduction. During tumor development, invasion and metastasis, there are significant biomechanical influences on various aspects such as tumor angiogenesis, interactions between tumor cells and the extracellular matrix (ECM), interactions between tumor cells and other cells, and interactions between tumor cells and the circulatory system and vasculature. The tumor microenvironment comprises a complex interplay of cells, ECM and vasculature, with the ECM, comprising collagen, fibronectins, integrins, laminins and matrix metalloproteinases, acting as a critical mediator of mechanical properties and a key component within the mechanical signaling pathway. The vasculature exerts appropriate shear forces on tumor cells, enabling their escape from immune surveillance, facilitating their dissemination in the bloodstream, dictating the trajectory of circulating tumor cells (CTCs) and playing a pivotal role in regulating adhesion to the vessel wall. Tumor biomechanics plays a critical role in tumor progression and metastasis, as alterations in biomechanical properties throughout the malignant transformation process trigger a cascade of changes in cellular behavior and the tumor microenvironment, ultimately culminating in the malignant biological behavior of the tumor.

## 1 Introduction

Cancer, as the leading cause of global mortality, remains an elusive foe despite significant global efforts in basic research, clinical practice and understanding of its complex mechanisms. While progress has been made in the development of anti-cancer drugs, diagnostic tools and treatment techniques, the elusive nature of most cancers poses substantial obstacles to achieving a complete cure. This is particularly evident in China, where both cancer incidence and mortality rates have skyrocketed in recent decades, creating a profound sense of fear and urgency.

The complexity and uniqueness of cancer require multidimensional approaches for diagnosis, treatment and research. In this context, biology is emerging as a key discipline that is intricately linked to the understanding and management of this formidable disease. The interplay between mechanical factors and other physical, chemical and biological elements is a crucial component in unravelling the mystery of cancer. By integrating various theoretical and experimental methods from mechanics and biology into the field of tumor biomechanics, we can explore the intricate mechanical dynamics underlying cancer at molecular, subcellular, cellular, cell group, tissue, organ, system and human body scales. Such investigations aim to elucidate the mechanical properties of cancer cells and tumor tissues, as well as the influence of the tumor microenvironment on their growth and progression. Equally important is the translation of these mechanistic insights into practical applications for clinical cancer diagnosis and treatment.

Fundamentally, with a comprehensive and interdisciplinary approach, through in-depth research on cancer biomechanics, we have the potential to revolutionize current methods of cancer diagnosis and treatment, thereby further enhancing cancer cure rates and improving people’s quality of life (1, 2).

## 2 Application of single-cell sequencing in cancer research

Single-cell sequencing, as a groundbreaking technology for genomic transcriptomic, and epigenomic analysis, differs from traditional sequencing methods that rely on bulk samples and provide an average representation. It enables the study of individual cells, capturing the intricacies of cellular heterogeneity. Conventional sequencing approaches often overlook the diverse cellular composition within a sample, resulting in an incomplete understanding of complex biological processes. In contrast, single-cell sequencing technology revolutionizes this scenario by revealing invaluable information about cellular heterogeneity that remains inaccessible when studying mixed samples.

Tumor cells, in contrast to normal cells, display distinct pathological and metabolic alterations, leading to the emergence of diverse tumor cell lineages and contributing to tumor cell heterogeneity. By providing high-resolution detection and analysis at the single-cell level, single-cell sequencing unravels the functional and physiological states of each cell, facilitating the accurate identification and characterization of different cellular subsets within tumor tissues (3).

### 2.1 Revealing single-cell genetic heterogeneity in cancer development

Whole exome sequencing was performed in mice, and repeated sampling was conducted by extracting cells from mice xenografted with lung adenocarcinoma tumor cells for single-cell transcriptome sequencing. Heterogeneity was observed in the nucleotide sequences of 50 tumor cells, including KRASG12D, and this heterogeneity was also present in all xenografted mouse cells (4).

Two cell lineages with distinct characteristics were observed in 12 patients with stage III-IV colorectal cancer by sequencing the primary, paracancerous and metastatic tissues; heterogeneity in DNA methylation and chromosome copy number variation was found in cells of different lineages, and there was a strong correspondence between the groups (5).

The role of integrin-mediated mechanical signaling in liver pathophysiology and homeostasis has been demonstrated by cre/loxP-mediated gene deletion of β1-intergin or siRNA mediated knockdown of β1-intergin. Meanwhile, in HCC patient samples, tumor sclerosis was positively correlated with β1-intergin expression (1, 6).

### 2.2 Revealing tumor heterogeneity

Heterogeneity, a prominent hallmark of malignant tumors, encompasses the genetic alterations that occur during the processes of gene replication, transcription and translation. These alterations give rise to distinct cell populations within tumors, resulting in phenotypic discordance.

Tumor heterogeneity manifests itself at four levels: inter-tumor variance, intra-tumor variance between different lesions, intra-tumor variance within different regions and intra-tumor variance between different cells within the same region. In a study of patients with myeloproliferative neoplasms, 82 bone marrow cells and 8 normal oral epithelial cells were randomly selected for single-cell whole-exome sequencing. The results showed heterogeneity in gene expression profiles between tumor cells and normal cells (7).

Research has shown that soft substrates can maintain cell differentiation, while rigid substrates(adsorbed on a single collagen film in a rigid culture dish) can lead to a dedifferentiated phenotype and continuous proliferation of liver cells. Stiffness increases the expression of genes encoding cytoskeleton regulatory proteins, activates integrin FAK/Src mechanical signaling, leading to gene transcription and new phenotypes of liver cells in vivo (8–10).

### 2.3 Studying gene sequence differences between different tumor subtypes

To investigate genetic variations in different tumor subtypes, single-cell transcriptome sequencing was employed to analyze marrow cells from 16 acute myeloid leukemia patients and 5 healthy donors. This analysis successfully yielded a detailed and comprehensive atlas of tumor cells and normal cells of various types and differentiation states. Notably, multiple subclones with distinct genetic profiles were identified within a single patient sample (11).

In another study, single-cell transcriptome sequencing was performed on cells derived from IDH-A and IDH-O tumors, and their sequencing results were then compared with data from The Cancer Genome Atlas (TCGA), revealing significant differences in the tumor microenvironment between IDH-A and IDH-O tumors (12).

### 2.4 Identifying immune cell subsets in the tumor

Researchers also conducted an investigation into the identification of immune cell subsets within tumors. By utilizing single-cell transcriptome sequencing, they analyzed 36,424 cells from 13 cases of prostate tumors. This analysis confirmed the capacity of cancer cells to modulate T-cell transcriptomes (13). Similarly, single-cell transcriptome sequencing was employed to examine T cells in peripheral blood tumor tissue and adjacent normal tissue from patients with hepatocellular carcinoma. The findings of this analysis uncovered the presence of abundant suppressor T cells and dysfunctional CD8+ T cells in tumor tissue. In addition, the analysis of TCR data revealed a substantial population of senescent T cells within the liver cancer, shedding light on the mechanisms of immune evasion by tumor cells (14).

### 2.5 Studying the occurrence and development of tumor

In this study, the scTrio-seq technique was used to examine 25 cancer cells in liver tissue from a patient diagnosed with liver cancer. Among the analyzed liver cancer cells, a smaller subset, known as subset I, showed a higher frequency of DNA copy number variations and increased levels of DNA methylation. These characteristics suggest that subset I cells may have a greater propensity to evade detection by the patient’s immune system (15).

By employing single-cell whole-exome sequencing, a comprehensive analysis of tumor cells derived from patients with bladder cancer identified 21 pivotal genes exhibiting tumor-specific mutations. Notably, the co-occurrence of mutations in ARID1A, GPRC5A and MLL2 was found to play a significant role in cancer development (16).

Single-cell sequencing, has revealed that certain unique tumor cells exhibit structural heterogeneity and experience significant fluctuations in their mechanical characteristics. This dynamic behavior plays a crucial role in tumor metastasis (17), facilitating the conversion of epithelial cells to mesenchymal cells through a process known as epithelial-mesenchymal transition(EMT) (18). The loss of E-cadherin leads to the dynamic change of the physical and mechanical properties of these cells.

## 3 The matrix metalloproteinase family

The matrix metalloproteinase (MMP) family comprises several members, including MMP-2, MMP-7, MMP-9, and MMP-12, which contribute to the assembly of the tumor matrix metalloproteinase.

MMPs are synthesized and secreted by both connective and tumor tissues and form a critical proteolytic system responsible for the degradation of the extracellular matrix. Analysis of gene expression changes in breast cancer, from normal tissue to precancerous lesions and invasive ductal carcinoma, unveiled a significant upregulation of MMP-2, MMP-11 and MMP-14 within the stroma rich in matrix metalloproteinases. This observation suggests that the tumor microenvironment actively participates in tumorigenesis even before infiltration by tumor cells (19).

In ductal carcinoma in situ, MMP-2 and MMP-9 exhibited high expression levels compared to normal and hyperplastic tissue (20). Other studies have also reported increased expression of MMP-1, MMP-2, MMP-3, MMP-9 and MMP-11 (21, 22). The heightened expression of MMP-1 and MMP-12 in aggressive tumor stroma is associated with a poorer prognosis.

## 4 The biomechanical relationship between the cell and the ECM

The extracellular matrix consists mainly of collagen, fibronectin, integrin, laminin and matrix metalloproteinase laminin. Collagen serves as the primary architectural support for the mechanical properties of tissues (23) and influences the stiffness of the extracellular matrix. Changes in cellular hardness are associated with tumor progression (24). Among the collagen isoforms, collagen I, III and IV are the most abundant. Collagen I, in particular, forms an important barrier structure during the invasion of tumor cells, and upregulation of its expression can increase the stiffness of tumor tissue. Additionally, tumor cells must migrate through the basement membrane, which is enriched in collagen IV and fibronectin.

Increased tissue stiffness contributes to the mechanical stiffness observed in malignant tissue compared to normal or benign tissue. Increased cross-linking of Collagen I leads to increased stiffness of malignant tissue, which is one of the reasons why malignant tissue is “Stiffer” than normal or benign tissue at a macroscopic level. Studies indicate that the tissue strain rate (SI) in malignant prostate cancer (PCA) exceeds that of benign prostatic hyperplasia (BPH), meaning that tumor tissue has greater stiffness than non-tumor tissue. In a confined space, the tension generated by the excessive proliferation of tumor cells pushing against each other also increases the macroscopic stiffness of the tumor tissue. When a physician applies pressure to a tumor using a finger or an ultrasound probe, the feedback received is not related to the physical texture of the tumor, but rather to its biomechanical behavior (25). In addition, high-grade tumors have greater stiffness than low to intermediate grade tumors, indicating a positive correlation between tissue stiffness and tumor grade.

However, the overall hardness of the tumor does not necessarily correspond to its consistency as a whole. MMP-2-mediated degradation of Collagen I may be responsible for the decrease in micromechanical properties of cancer tissues. Advanced and metastatic tumors exhibit significantly lower collagen expression compared to low-intermediated and non-metastatic tumors, and this decrease is dependent on MMPs. The expression of MMPs peaked in the low-to-intermediate grade group, while the high-grade tumors had metastasized and the decrease or absence of Collagen resulted in decreased secretion of MMPs (26). The loss of cytoskeleton elements and related proteins and the remodeling of cytoskeleton may result in the soft texture of tumor cells under microcosmic conditions. Research indicates that the Young’s modulus of PCA tissue is lower than that of BPH tissue, and this difference increases with increasing pathological grade and metastasis. The decrease in Young’s modulus serves as an indicator of high-grade malignancy and facilitates metastasis. This suggests that highly malignant tumor cells or cancer stem cells (CSCs) have lower cellular stiffness compared to low malignant or non-CSCs, making them softer than healthy cells. Therefore, there exists an inverse correlation between cellular stiffness and malignancy (24). Additionally, a triangular relationship can be observed between cell stiffness, phagocytic capacity and malignancy of tumor cells. Tumor cells characterized by high phagocytic capacity are softer and show similarities to CSCs, demonstrating increased carcinogenicity in mouse models (24, 27).

Changes in collagen I concentration have a direct effect on cell morphology, adhesion, mechanical properties, invasiveness and sensitivity to apoptosis. AFM (atomic force microscopy) results have shown that tumor cells have the lowest Young’s modulus at lower concentrations of collagen I. Moreover, tumor cells demonstrate enhanced invasive potential at lower collagen I concentrations compared to higher concentrations. This phenomenon facilitates the acquisition of unlimited proliferative capabilities in cancer cells.

Macroscopically, malignant tissue is harder than normal or benign tissue, while microscopically, the Young’s modulus of malignant tissue is lower than that of benign tissue. This confirms that malignant tissue is softer than benign tissue. For instance, untreated chronic liver disease can lead to liver fibrosis and the development of cancer, resulting in stiffening of the liver. In response to this stiffness, both hepatic stellate cells and portal vein fibroblasts, which are sensitive to mechanical cues, undergo permanent activation and promote the formation of myofibroblasts. These myofibroblasts contribute to the production of additional matrix components (1). Furthermore, the decrease in collagen content and subsequent reduction in Young’s modulus within the tumor cells themselves contributes to microscopic detection results that reflect lower values for malignant tumors compared to benign tumors (28).

MMP-2-mediated degradation of collagen I plays a critical role in the reduction of micromechanical properties in cancer tissues. The expression of collagen is notably lower in advanced and metastatic tumors compared to low to intermediate grade and non-metastatic tumors, and this decrease is dependent on MMP activity. The expression of MMPs reaches its peak in low-to intermediate-grade tumors, while high-grade tumors metastasized and had significantly reduced amounts of collagen. Consequently, the secretion of MMPs, which primarily degrade collagen in tumor tissue, is also diminished. In general, the reduction or absence of collagen leads to a decrease in MMP secretion (26).

Changes in extracellular matrix stiffness affect the differentiation state of cells. Research has shown that the softness of the substrate plays a critical role in cell differentiation and the soft matrix keeps the cells in a differentiated state, whereas rigid substrates, such as collagen films adhered to inflexible Petri dishes(simulated a stiff extracellular matrix), result in a dedifferentiated phenotype of hepatocytes that proliferate continuously. The substrate stiffness actively promotes the expression of genes involved in regulating the cell cytoskeleton, initiating mechanistic signals via integrin-FAK/SRC activation, and subsequently triggering gene transcription, ultimately culminating in the emergence of a distinct hepatocyte phenotype within the organism (8–10).

Matrix metalloproteinases (MMPs) are responsible for the consolidation and remodeling of the extracellular matrix. Mechanical transduction, the process through which biological forces impact cellular behavior, profoundly influences cellular function and status. This transduction mechanism involves intricate interactions among various cellular components, including mechanical receptors located on the cell membrane, associated protein complexes, and mechanical sensors. Biological forces originating from the extracellular matrix are transmitted into the cell interior via mechanistic receptors such as integrins at cell-extracellular matrix junctions, E-cadherin at cell-cell contacts, ion channels activated by mechanical tension, and receptor tyrosine kinases (29). These changes in molecular sensing facilitate amplification and transduction of the relevant protein complex.

Subsequent investigations have provided additional insights into the significance of integrin-mediated mechanical signaling in liver pathophysiology and homeostasis. This understanding has been achieved through the use of CRE loxP-mediated gene deletion of β1-integrin or the application of siRNA to knock down β1-integrin expression. Furthermore, a positive correlation has been observed between the expression of β1-integrin and tumor sclerosis in samples of hepatocellular carcinoma(HCC) (6).

**Figure 1:**
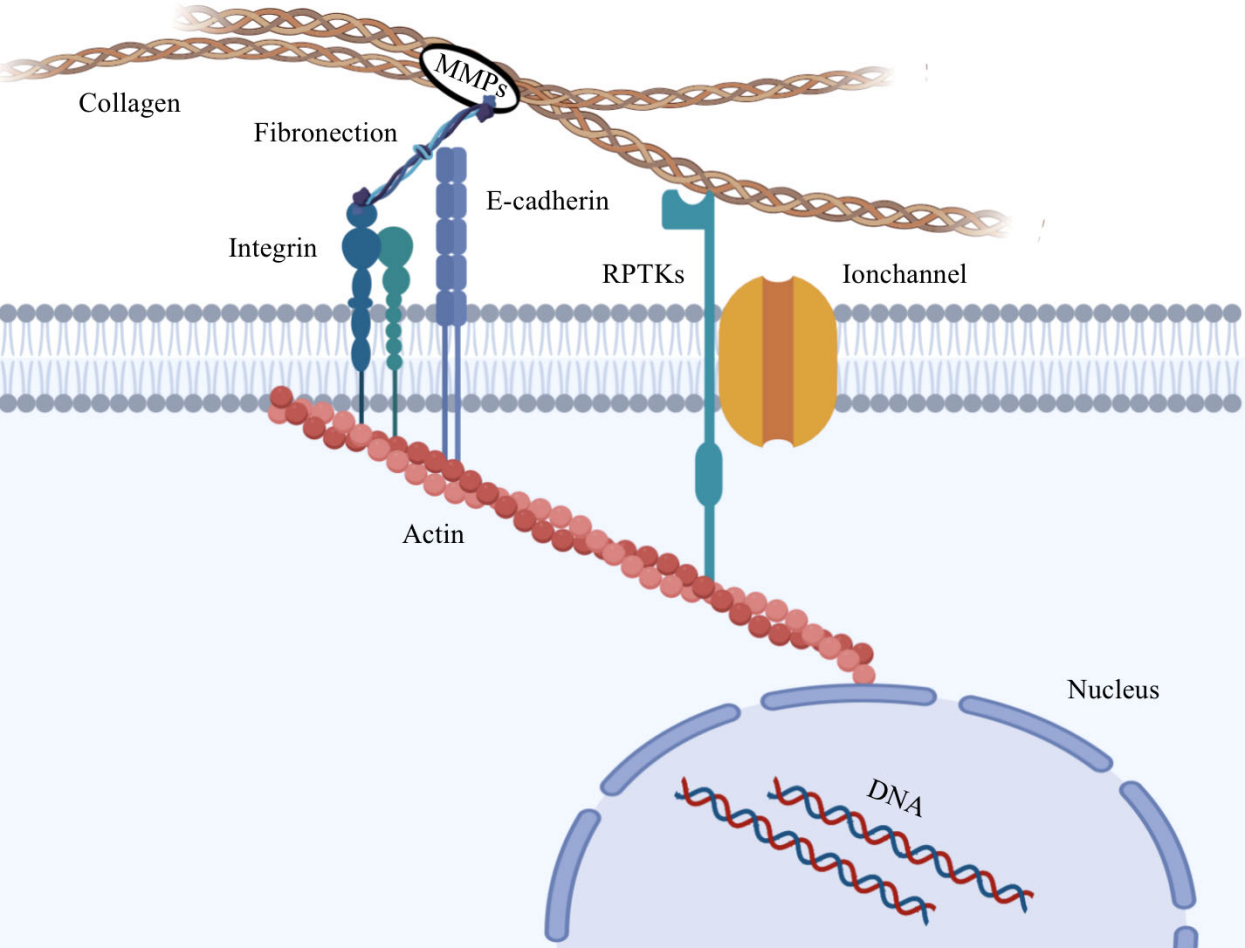
Diotransmission pathway between tumor cells and extracellular matrix. Collagen in the extracellular matrix is hydrolyzed by MMPs, which alters the biomechanical properties of the extracellular matrix. The biological forces in the ECM are transmitted to the cytoskeleton through mechanical conducting elements on the cell membrane, and then transmitted to the nucleus to cause heterogeneity of gene expression.

Biomechanical transmission relies on the intricate network of the cytoskeleton, which serves as a crucial determinant of cell shape and integrity (30). In addition, the cytoskeleton affects mechanical transduction, migration properties and even the contractile forces generated by cytoskeleton-binding proteins, which in turn remodel the cytoskeleton and affect cell stiffness (31–34). In particular, during cancer development, cytoskeletal proteins undergo significant changes that are closely linked to tumor progression (35, 36). These proteins form a dynamic network connecting the cell membrane to the nucleus, with myosin contractility and myosin motor playing a critical role in cytoskeletal tension. Elevated cytoskeletal tension leads to increased adhesion, which is facilitated by the attachment of actin and intermediate filaments to the nephrin protein found on the nuclear membrane. This connection allows external forces to reach the nucleus, which is coupled to the cytoskeleton through the nuclear skeleton (37). As a result, the nucleus receives forces from the cytoskeleton and converts them into biochemical signals that influence the cellular gene expression process. Biomechanical forces induce the opening of nuclear pores and enhance the nuclear transport of proteins, subsequently impacting gene expression (38–40). **Figure 1** visually illustrates this phenomenon.

## 5 Changes in biomechanical properties of tumor cells

In the context of tumor cells, E-cadherin plays a pivotal role in the establishment of adhesion junctions within epithelial cells. To initiate adhesion junctions, the extracellular portion of cadherin molecules interacts with identical cadherin molecules on neighboring cells, while the cytoplasmic tail binds to p120 and β-catenin proteins (41). Subsequently, β-catenin interacts with α-catenin, which possesses an actin-binding domain. This linkage physically connects adherens junctions to the actin cytoskeleton, collectively triggering changes in cytoskeletal structure and adaptive cytoskeletal sclerosis as integral components of the mechano-chemical signaling response (42).

It has been found that the force exerted on the cell surface can be transmitted directly to the chromatin through the cytoskeleton and nuclear proteins, and the stretching of chromatin directly activates gene expression. In vivo, cells are subjected to forces of different modes. However, it is worth noting that stresses of the same magnitude but with different modes of transmission can lead to different cellular responses. These responses include different degrees of nuclear chromatin elongation, resulting in diverse levels of gene expression. The study found that the expression of genes could be detected after 15 seconds of continuous stretching of chromatin. Gene expression is directly related to the direction, magnitude, and duration of the applied force. When subjected to the same force, gene expression level are lowest in the direction parallel to the cell’s long axis and highest in the direction perpendicular to the long axis. Within the physiological range, gene expression level demonstrate a positive correlated with the external force exerted. Gene expression level increase with prolonged duration of loading and reach a peak after 90 minutes of loading before stabilizing. Experiments have shown that mechanical signals can induce the expression of the transduced Dihydrofolate reductase genes, as well as the expression of endogenous EGR-1 genes (43). The heterogeneity of tumor cells, as evidenced by variations in gene expression, can be identified using single-cell sequencing techniques.

Certain tumor cells frequently exhibit a wide range of structural heterogeneity and diverse mechanical properties (17), enabling their activation during tumor metastasis and facilitating the transition of epithelial cells to mesenchymal cells through a phenomenon called epithelial-mesenchymal transition (EMT) (18). A critical aspect of this process is the loss of E-cadherin, which directly affects the physical and mechanical properties of cells.

Numerous studies have consistently demonstrated that the frequent loss of E-cadherin in human epithelial cancers. Furthermore, E-cadherin restoration has been shown to reduce cancer cell proliferation, while its disruption promotes cell proliferation in three-dimensional(3D) culture models (44). Epithelial-mesenchymal transition plays an important role in metastasis and typically involves remodeling of the actin cytoskeleton, morphological changes and cell softening (45). Highly metastatic cancer cells acquire a mesenchymal phenotype characterized by low density and disorganized stress fibers (46). The actin cytoskeleton plays a critical role in the formation of lamellipodia (actin projections at the leading edge of the cell) and invadopodia (critical structures for maintaining the high migratory and invasive ability of tumor cells), making it essential for tumor cell invasion and metastasis (24).

Cells shed from the primary tumor and acquire a dynamic phenotype that enables them to invade surrounding tissue and express matrix metalloproteinases (MMPs). This creates space for tumor cells to metastasize (35). During migration through confined spaces, the stress fibre network undergoes remodeling, potentially leading to cellular softening (24, 47). Importantly, the invasive phenotype of tumor cells can be transmitted across multiple generations, thereby enhancing their proliferative and spreading capacities.

## 6 The biomechanical relationship between cells and the vasculature

As solid tumors grow in volume, the increased tension in the tissue not only affects tumor growth, but also deforms tumor blood vessels. Throughout tumor progression, the delicate balance between pro-angiogenesis and anti-angiogenesis is disturbed, triggering the activation of the angiogenesis switch (48). As tumor tissue grows, its volume continues to increase, leading to increased tension within the tissue. This in turn causes deformation of tumor vessels and affects tumor growth (42, 49). These forces present in the tumor microenvironment can be categorized as either solid stress or fluid stress, both of which activate signaling pathways critical for proliferation, survival, invasion and metastasis (50).

Solid stress is primarily derived from the non-liquid components of the tumor, including cancer cells, various host cells and the extracellular matrix (ECM) (42, 49). Tumor growth leads to the accumulation of solid stress within the tumor, and as the density of cells and matrix increases, solid stress significantly escalates. When the tumor tissue becomes stiffer compared to the surrounding normal tissue, solid pressure accumulated, resulting in the acquisition of invasive properties (50). The accumulation of compressive stress during tumor growth induces invasiveness in cancer cells. Experiments that simulate solid stresses in tumors through compression have shown that they can alter cancer cell adhesion and migration (51), increasing TME stiffness stimulates the secretion of activin a, a strong metastatic cytokine in cancer-associated fibroblasts, and matrix-secreted activin a induces ligand-dependent CRC epithelial cell migration and EMT. These findings suggest that mechanical forces in the TME promote aggressive behavior of cancer cells, including proliferation, migration, invasion and sphere development, ultimately promoting the aggressive behavior of tumor cells (50). Furthermore, as tumor and stromal cells proliferate and migrate through the ECM, growth-induced stiffness is generated and propagated through this matrix (42, 52). Studies have revealed a strong correlation between galectin-1 overexpression in pancreatic cancer and angiogenesis. Increased contraction of fibroblasts and other stromal cells within tumors induces tissue tension, which subsequently affects the growth of tumor vessels. The growth and expansion of vascular tissue is closely linked to and controlled by tissue contraction, which causes endothelial cells to align along the direction of tension (42, 53, 54).

There exists a significant difference in elastic modulus between benign and malignant tumor tissue. Alterations in the biomechanical properties of the extracellular matrix lead to changes in the cytoskeleton. The stiffness of tumor cells increased, the harder the tissue was. Accelerated transmission of shear waves corresponds to a higher Young’s modulus and increased cellular stiffening. As a result, the elastic modulus of the cells is increased, F-actin fiber bundle multiaxial stretching cell, leading to cell deformation and creating favorable conditions for cell passage through endothelial junctions. Simultaneously, increased ECM stiffness increases endothelial permeability in vivo (55). Subsequently, tumor cells adhere to the vessel wall, allowing blood flow to transport these cells to distant sites where secondary tumors form within the local tissue. As normal epithelial cells transition from a normal state to a malignant and metastatic state, the levels of F-actin continuously increase, leading to cell deformation (56). Interestingly, breast cancer cells predisposed to bone metastasis have higher levels of F-actin than tumor cells predisposed to brain metastasis (57). This suggests a link between tumor cell mechanics and the propensity to metastasize to specific organs (24). When F-actin is compromised, the cell’s elastic modulus decreases, rendering it softer (58).

Fluid stress within the tumor microenvironment includes the collective force exerted by various fluid components of the tumor, including microvascular fluid stress, interstitial fluid stress and shear stress. These stresses arise from the flow of blood and interstitial fluid (42, 49). The presence of solid stress and the accumulation of fluid in the interstitial space contribute to high levels of interstitial fluid stress (59, 60). This interstitial fluid stress can guide tumor cell migration through autocrine CCR7 signaling (61). Even low levels of sustained fluid shear stress can significantly impact the adhesive properties of epithelial ovarian cancer cells at different stages of progression (62). As mentioned earlier, interstitial fluid stress within the tumor microenvironment can direct cell movement and promote tumorigenesis (50).

**Figure 2:**
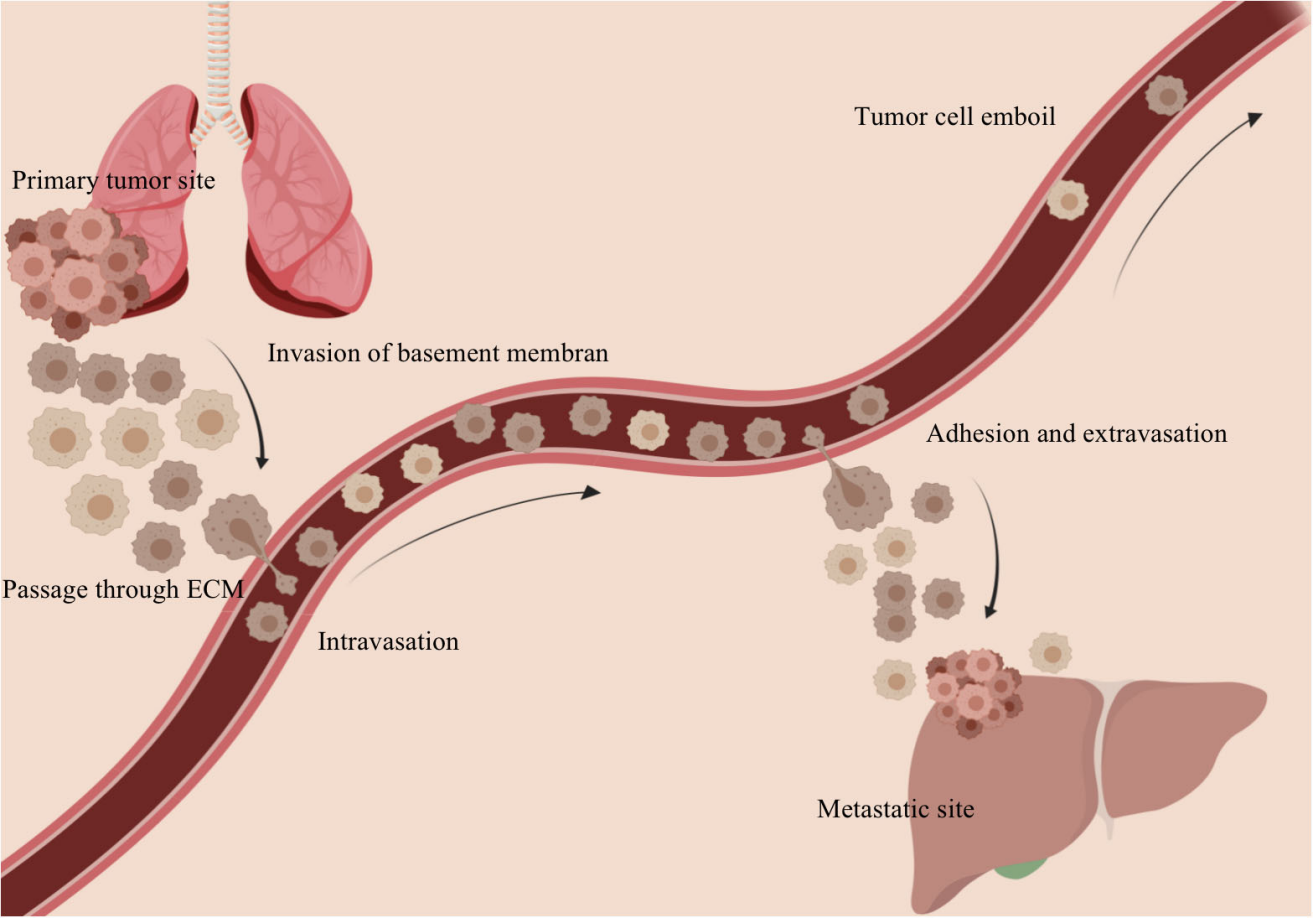
Tumor cells metastasize and colonize through the vasculature. The primary tumor tissue invades the basement membrane and penetrates the extracellular matrix into the vasculature. With blood flow and under the action of fluid shear stress, secondary lesions are formed in the appropriate remote location.

Fluid shear stress affects signaling cascades that influence endothelial cell morphology, thereby triggering remodeling of the vascular network (63) and inducing an invasive phenotype. The abnormal structure of tumor microvessels leads to increased geometric and viscous resistance within the blood flow (49), as well as mechanical forces within the tumor microenvironment caused by vascular compression, limiting tumor perfusion (64–66). Vascular compression, together with the highly tortuous and disorganized arrangement of tumor vessels, results in slow blood flow, hypoxia and tumor cell heterogeneity (67). Hypoxia can induce early changes in gene expression, and proteome, controlling anabolic switches in central metabolism to affect metabolism and promote malignant progression, form a more aggressive and difficult to treat tumor phenotype (68, 69). The abnormal tumor microenvironment exerts selective pressure, forcing tumor cells to dynamically adapt (42, 70). Proliferation, division and metastasis occur under the influence of selective pressure in this harsh environment (71). Hydrodynamic shear stress has been shown to facilitate the conversion of circulating tumor cells (CTCs) into distinct, less rigid cancer stem cells in the bloodstream (50, 72–74). During infiltration and extravasation, tumor cells undergo metaplasia into endothelial cells, during which the tumor cells and their nuclei become softened. At the same time, these less rigid cancer stem cells mimic endothelial cells and facilitate tumor metastasis (see **Figure 2**) (75, 76). The MDA-MB-231 cells that metastasized to the lung exhibited greater softness and migratory ability compared to both the CTCs derived from the original tumor cells and the parental tumor cells (24, 77).

Studies have shown that shear stress induces liver sinusoidal endothelial cells (LSECs) to produce mechanical signals and release vascular secretory factors, thereby contributing to the growth and maintenance of liver function in normal physiological conditions (78). In the early stages of liver fibrosis, LSEC-dependent angiogenesis may lead to ECM stiffening, influencing the activation of hepatic stellate cell (HSC) and disease progression. The angiogenesis driven by LSECs causes the condensation of collagen fibers, and the resulting force response from collagen remodeling activates HSCs (79). Therefore, under pathological conditions, the biological forces present in the liver promote the development of diseases through mechanical transduction and the activation of LSECs.

Recent studies have unveiled the significant impact of modifying the biomechanics of the extracellular matrix on tumor development and vascular phenotype. Researchers have observed that nonenzymatic glycosylation, which increases three-dimensional collagen stiffness without altering matrix structure, leads to increased angiogenic growth and vessel branching density of endothelial cell spheroids in vitro. These alterations in matrix stiffness contribute to the formation of a tumor-promoting blood vessel phenotype (55). More specifically, an increase in stromal stiffness corresponds to an enhanced vessel growth response and increased vessel branching density (80–84). In addition, the presence of a stiffer matrix impairs barrier function and disrupts the localization of endothelial E-cadherin, leading to an increase in vascular permeability.

An intriguing observation is that the alterations in vascular phenotypes and the heightened angiogenic responses are reliant on the upregulation of MMP (matrix metalloproteinase) activity, particularly MMP-1. Activation of MMP-1 is dependent on cell contractility and the stiffness of the surrounding matrix (85). This discovery highlights the crucial role of MMPs in promoting angiogenesis (42, 55). In response to shear stress, VEGFR2 undergoes rapid induction and nuclear translocation followed by ligand-independent phosphorylation, leading to activation of MAPK, PI3K, and Akt signaling pathways, which are involved in promoting angiogenesis (86–88).

Two commonly used indices to quantify stiffness are Young’s modulus (E) and shear modulus (g), which are related by the equation 3G = E.

When tumor cells enter the bloodstream, their trajectory is affected by several biological forces, including shear and viscous (internal friction) forces. Shear force (τ) is generated on the adjacent surface near the blood flow due to the different velocities and viscosity (η) of the fluid. The magnitude of the viscous force (F) is proportional to the contact area (s) and the velocity gradient (dv/dx) at the contact, expressed as f = ηsdv/dx. τ = f/s denotes the viscous force acting per unit area, where γ = d γ/dt = dv/dx represents the shear rate or the rate of change of shear strain with time. In biomechanics, Newton’s law of viscosity is usually formulated as τ = ηγ. However, blood does not strictly obey Newton’s law of viscosity due to its non-Newtonian fluid behavior, characterized by variable viscosity. As a result, shear stress is not directly proportional to shear rate.

Shear stress has a profound effect on the movement of circulating tumor cells (CTCs), affecting both their parallel and rotational movements. This in turn influences the direction of cell migration and their receptor-ligand adhesion. Furthermore, shear stress plays a role in guiding CTC migration towards the vessel wall. Studies have shown that when tumor cells are exposed to a dynamic flow environment, the Young’s modulus decreases and the cytoskeletal structure changes. Changes in the cytoskeleton subsequently lead to changes in the mechanical properties of the cell. By establishing a relationship between the fluid flow environment and cell structure/mechanical properties, this study has provided new insights into the mechanical behavior of tumor cells under these conditions.

## 7 The biomechanical relationship between cells and target organs

Metastasis represents an extraordinary journey undertaken by tumor cells, during which they encounter diverse mechanical cues and undergo passive and/or active modifications in their cytoskeleton and biomechanics properties. This adaptive process empowers them to thrive within distinct tumor microenvironments throughout different stages of metastasis (24).

Results from transwell invasion experiments show that high levels of collagen I significantly inhibit the invasiveness of malignant tumor cells. In addition, it was observed that tumor cell apoptosis was more pronounced in regions with higher collagen concentrations, suggesting that elevated collagen levels increase the sensitivity of tumor cells to apoptosis.

Collagen I serves as a primary adhesion target for tumor cells and acts as a barrier to cell metastasis. However, for tumor cells to navigate through the collagen I-rich extracellular matrix (ECM), they first need to adhere to collagen I. This initial adhesion event triggers the secretion of matrix metalloproteinases (MMPs) and other hydrolytic proteins, resulting in a decrease in Young’s modulus at the microscopic level within advanced tumor tissue.

During local invasion, tumor cells at the invasive front exhibit softer characteristics compared to those in the central region (89). In the context of non-small lung cancer cell migration, leader cells display upregulated expression of mesenchymal markers such as snail and vimentin. These leader cells are softer and less adhesive compared to follower cells (90). The aforementioned study proposes two potential mechanisms to explain the reduction in cell stiffness observed in metastatic tumor cells. Firstly, highly invasive tumor cells tend to have lower stiffness than tumor cells with limited metastatic capacity (91). Within primary tumors there is mechanical heterogeneity between cells. The advantage of softer tumor cells lies in their ability to dissociate from the tumor mass and invade the surrounding stroma, facilitating metastatic spread. These findings suggest that the lower stiffness of invasive tumor cells is the result of an active selection process in which cell stiffness and invasiveness are inversely related. Secondly, both soft and hard tumor cells in primary tumors can spread and invade. When disseminated tumor cells navigate through dense tumor stroma or tight endothelium, they need to soften their cytoskeleton to achieve high deformability to successfully traverse endothelial gaps (57, 75). In addition, these two mechanisms may also act synergistically during tumor metastasis.

Research has shown that soft breast tumor cells of certain classifications exhibit greater stemness and tumorigenic potential compared to their stiff counterparts (92, 93). The softness of the cells prevents cytotoxic T lymphocyte(CTL)-induced pore formation, enabling them to evade CTL-mediated cell killing. Therefore, soft cells possess a greater ability to evade eradication by the immune system and are more likely to colonize target organs and subsequently establish new tumor sites (94). Furthermore, a clear correlation has been observed between cell stiffness, phagocytosis and tumor cell malignancy. Tumor cells with high phagocytic capacity tend to have softer characteristics resembling cancer stem cells(CSCs), exhibiting increased carcinogenicity in mouse models (27).

**Figure 3:**
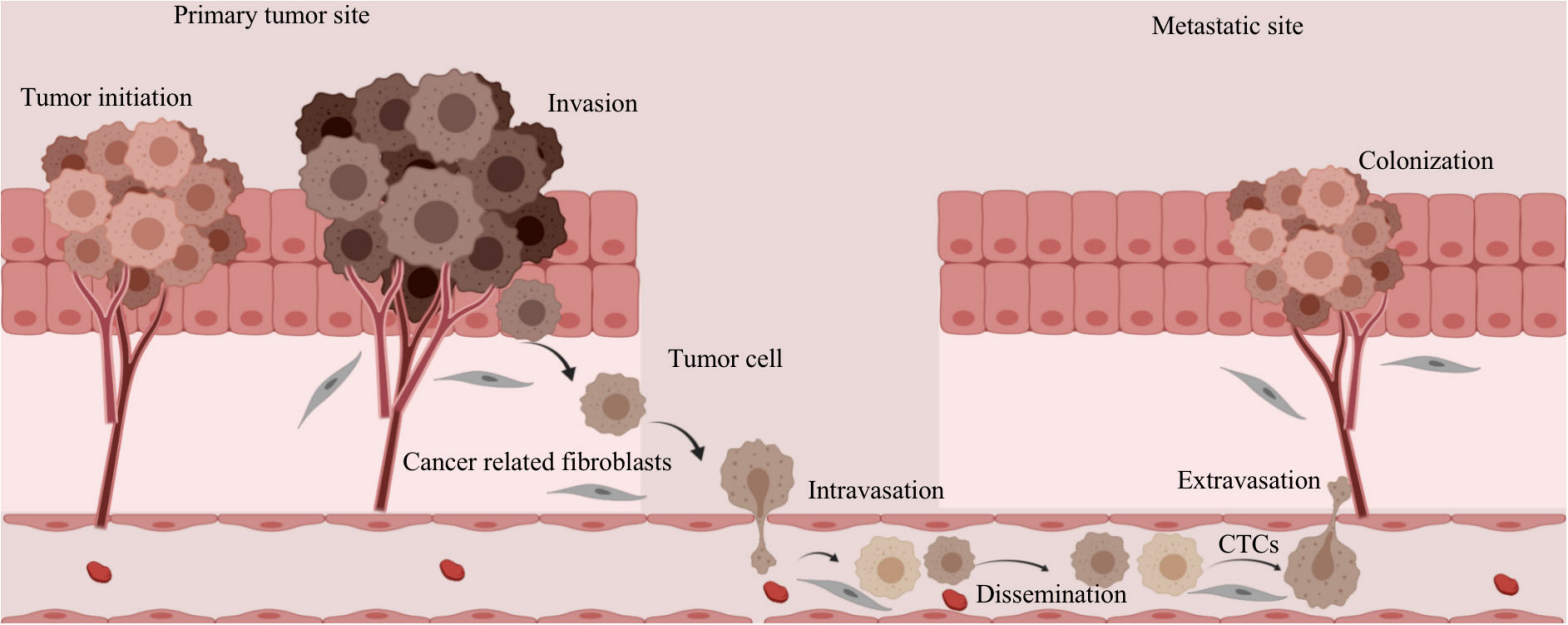
Changes in tumor stromal cells during metastasis and invasion of tumor cells. The metastatic tumor cells are accompanied by fragments of tumor stromal cells that enter the circulating blood, which increases the viability of the tumor cells and provides a favorable environment for their implantation and initial growth.

Metastatic clones, as evidenced by numerous post-mortem studies, do not distribute randomly (95). Two prevailing hypotheses shed light on the process of cancer metastasis. The “seed and soil” hypothesis posits that cancer cells tend to migrate to more favorable environments (96). In particular, collagen I provides an ideal environment for the growth of malignant tumor cells by allowing them to adhere to it, thereby inhibiting tumor migration. The “mechanical force” hypothesis suggests that the sites of displacement are determined by patterns of blood flow and shear forces (97). For example, when metastatic tumor cells accompany debris from tumor stromal cells into the bloodstream, they acquire greater viability and create a conducive environment for their colonization and initial growth (as shown in **Figure 3**) (98). In addition, cancer-associated fibroblasts play a role in the formation of distant metastatic sites by co-migrating with cancer cells and creating pathways within tissues, thereby facilitating invasion and migration processes (99, 100).

Blood flows back through the venous system to the heart and through the heart to the lungs for oxygen and through the arterial system to the organs. The capillary of each organ is a network of small blood vessels. Tumor cells entering vessels smaller than their diameter have a higher likelihood of being trapped by physical occlusion. To successfully form new metastases, tumor cells must penetrate blood vessels and colonize distant tissues. The interaction of blood flow patterns between primary and secondary lesions accounts for over 50% of tumor metastasis. The longer circulating tumor cells collide with the vessel wall in larger vessels and remain in a relatively dormant state, the greater the likelihood that they will adhere to the vessel wall and subsequently extravasate. The extent to which these cells remain in place and have sufficient residence time to extravasate and clone is influenced by local fluid shear forces. Therefore, an optimal shear stress condition and duration of cell residence along the vessel wall are required for successful extravasation and subsequent cell movement and invasion.

## 8 Discussion

The entire process of tumor cell initiation, development, metastasis, invasion and adhesion has been extensively studied. The mechanisms involved in tumorigenesis alter the properties of the extracellular matrix, including collagen, fibronectin, the integrin family, laminin and matrix metalloproteinase. These components are the primary contributors to extracellular-mediated mechanical changes and serve as key factors in the mechanical conduction pathway. Changes in the stiffness of the extracellular matrix allow biological force signals to be transmitted through the cytoskeleton to the nucleus via interactions with multiple mechanoreceptors on the cell membrane. This process affects gene expression and modifies the malignant phenotype of tumor cells (101). At the macroscopic level, tumor tissue exhibits increased stiffness and strain rate compared to normal tissue, indicative of heightened hardness. Conversely, tumor cells exhibit decreased Young’s modulus and stiffness, signifying a softer composition compared to healthy cells. This softer composition enhances the deformability, metastatic potential and invasiveness of tumor cells.

Cadherin, a critical component in the formation of adhesion junctions in epithelial cells, plays a crucial role in bridging the cytoskeleton and transmitting biomechanical signals between neighboring cells. Consequently, the cytoskeleton undergoes synchronized remodeling, leading to cell softening and the acquisition of a mesenchymal phenotype, which is advantageous for metastasis and invasion.

During tumor progression, the delicate balance between pro-angiogenesis and anti-angiogenesis is disturbed and the angiogenesis switch is activated (42, 48). This process results in increased tumor angiogenesis density. Under solid stress, tumor cells cross the endothelial barrier and invade the vasculature. Subsequently, under the combined influence of fluid stress and fluid shear stress, cellular structure undergoes further modification, increasing softness and facilitating escape from immune surveillance. Additionally, this modification promotes the dissemination of tumor cells in the bloodstream, dictates the trajectory of circulating tumor cells, and significantly affects adhesion to the vessel wall. Under suitable shear stress conditions, tumor cells can invade endothelial cells, culminating in the formation of new metastatic foci.

Tumor biomechanics plays a critical role in tumor progression and metastasis. This review primarily focuses on how external biological forces are perceived, amplified and transmitted to the cell’s interior. These forces are then transmitted to the nucleus, influencing gene expression and biological responses. The alteration of cellular mechanical properties during malignant transformation leads to changes in cell behavior and the overall tumor microenvironment. Ultimately, these changes determine the invasiveness and metastatic potential of the tumor.

## 9 Conclusion

Currently, most research on cancer primarily focuses on aspects such as familial genetics, genetic mutations, living environments, and lifestyle. Research into the impact of biomechanics on the onset and progression of cancer remains relatively limited. Delving deeper into the influence of biomechanics can not only enable earlier and more accurate detection of cancer development and tumor formation but can also broaden the discourse on factors contributing to cancer progression. This area of research is therefore of considerable theoretical and clinical value.

## Author contributions

LB: Writing – original draft. HK: Writing – review & editing. YJ: Writing – original draft. CW: Writing – original draft. LQ: Writing – review & editing. HS: Writing – review & editing. SD: Writing – original draft, Writing – review & editing.
